# Advancements in Myocardial Infarction Management: Exploring Novel Approaches and Strategies

**DOI:** 10.7759/cureus.45578

**Published:** 2023-09-19

**Authors:** Pranav Sachdeva, Kawanpreet Kaur, Saba Fatima, FNU Mahak, Muhammad Noman, Sowmya Manjari Siddenthi, Marvi Alais Surksha, Mishaal Munir, FNU Fatima, Syeda Salima Sultana, Giustino Varrassi, Mahima Khatri, Satesh Kumar, Mahir Elder, Tamam Mohamad

**Affiliations:** 1 General Medicine, Government Medical College & Hospital, Chandigarh, Chandigarh, IND; 2 Medicine, Jinnah Sindh Medical University, Karachi, PAK; 3 Medicine, Jinnah Postgraduate Medical Centre, Karachi, PAK; 4 Internal Medicine, Ziauddin University, Karachi, PAK; 5 Internal Medicine, Osmania Medical College, Hyderabad, IND; 6 Medicine, Ghulam Muhammad Mahar Medical College, Sukkur, PAK; 7 Medicine, Ghurki Trust and Teaching Hospital, Lahore, PAK; 8 Internal Medicine, Lahore Medical & Dental College, Lahore, PAK; 9 Medicine, Southern Medical College and Hospital, Chittagong, BGD; 10 Pain Medicine, Paolo Procacci Foundation, Rome, ITA; 11 Medicine and Surgery, Dow University of Health Sciences, Karachi, Karachi, PAK; 12 Medicine and Surgery, Shaheed Mohtarma Benazir Bhutto Medical College, Karachi, PAK; 13 Interventional Cardiology, Heart and Vascular Institute, Detroit, USA; 14 Cardiovascular Surgery, Wayne State University, Detroit, USA

**Keywords:** myocardial infarction, advancements management, novel approaches, strategies, cardiac care

## Abstract

In the landscape of healthcare, the management of myocardial infarction (MI) stands as a pivotal challenge and a critical juncture where advancements are reshaping the trajectory of patient care. Myocardial infarction, commonly known as a heart attack, remains a foremost contributor to global morbidity and mortality. Conventional management strategies have historically focused on rapid restoration of blood flow through revascularization techniques. However, the last decade has witnessed a profound transformation, with a burgeoning emphasis on precision medicine and innovative interventions. This contextual backdrop sets the stage for a deep dive into the realm of novel diagnostic modalities, spanning high-sensitivity biomarkers, advanced imaging techniques, and data-driven algorithms. These innovations facilitate not only early detection but also the stratification of patients, paving the way for individualized treatment plans. By targeting the underlying mechanisms of myocardial damage, these interventions hold the promise of attenuating the impact of MI and promoting cardiac regeneration. It examines the integration of telemedicine, wearable devices, and remote monitoring platforms, bridging the gap between patients and caregivers while enabling timely interventions. Additionally, the psychosocial aspects of MI recovery are explored, highlighting the integration of psychological support and lifestyle interventions to enhance long-term well-being. By exploring novel diagnostics, innovative therapies, and holistic patient-centered strategies, it underscores the collaborative efforts of medical practitioners, researchers, and technological pioneers in reshaping the trajectory of MI care. As we stand at the intersection of medical advancement and compassionate patient management, embracing these novel approaches promises a future where the impact of myocardial infarction can be mitigated, and lives can be extended and enriched.

## Introduction and background

Myocardial infarction (MI), commonly known as a heart attack, remains a significant global health concern, exerting a substantial burden on individuals, families, healthcare systems, and economies. As a leading cause of mortality and morbidity worldwide, MI demands continuous advancements in its management to improve patient outcomes and enhance the quality of life for those affected [1]. Over the past few decades, the landscape of MI management has witnessed remarkable transformations, driven by innovative approaches and novel strategies that have revolutionized the understanding, diagnosis, treatment, and prevention of this critical condition. This narrative review embarks on a comprehensive exploration of the recent advancements in myocardial infarction management, with a specific focus on delving into the intricacies of these novel approaches and strategies that are reshaping the way MI is managed on a global scale [2]. Myocardial infarction occurs when there is a sudden interruption of blood supply to a portion of the heart muscle, leading to irreversible damage and impaired cardiac function. The condition arises predominantly from the rupture or erosion of an atherosclerotic plaque within the coronary arteries, resulting in the formation of a blood clot that obstructs blood flow. The consequences of MI can range from mild discomfort to severe heart failure and death [2]. Despite substantial advances in medical knowledge and technology, the management of MI remains a complex challenge due to its multifactorial nature, encompassing genetic predisposition, lifestyle factors, metabolic imbalances, and an intricate interplay of molecular pathways. Historically, the primary goal of MI management has been rapid reperfusion of the affected coronary artery to restore blood flow and salvage jeopardized myocardium [1]. The introduction of percutaneous coronary intervention (PCI) and thrombolytic therapy marked significant milestones in the field, leading to improved outcomes and reduced mortality rates. However, these traditional approaches have certain limitations. For instance, thrombolytic therapy is associated with an increased risk of bleeding complications, and PCI might not always be feasible due to delays in seeking medical attention, logistical challenges, or anatomical complexities [3]. The last decade has witnessed a remarkable paradigm shift in the management of myocardial infarction, driven by a deepened understanding of its pathophysiology, groundbreaking technological advancements, and the application of precision medicine principles. This shift has given rise to a multifaceted approach that goes beyond the conventional boundaries of reperfusion therapy, aiming to optimize patient care through a combination of early diagnosis, targeted therapies, innovative interventions, and comprehensive rehabilitation strategies [2]. Advancements in diagnostic modalities have played a pivotal role in early MI detection, enabling timely interventions and improved patient outcomes. Traditional diagnostic methods, such as electrocardiography (ECG) and cardiac enzyme assays, continue to serve as cornerstones in MI diagnosis. However, recent years have witnessed the development of high-sensitivity cardiac troponin assays that allow for the early identification of myocardial injury, even in cases of subtle damage [1]. These assays provide clinicians with a finer resolution in assessing cardiac biomarker levels, facilitating quicker decision-making and risk stratification.

Additionally, advanced imaging techniques, such as cardiac magnetic resonance imaging (MRI) and computed tomography (CT) angiography, offer a non-invasive window into the myocardium's structure and function. Cardiac MRI provides insights into myocardial viability, infarct size, and left ventricular function [2]. CT angiography, on the other hand, enables the visualization of coronary artery anatomy, helping to identify culprit lesions and guiding treatment decisions. The integration of these innovative diagnostic tools contributes to the precise characterization of the extent and severity of myocardial damage, enabling tailored therapeutic interventions. Precision medicine has emerged as a transformative approach in myocardial infarction management, emphasizing the customization of treatment strategies based on patients' unique characteristics. Genetic profiling and pharmacogenomics play a pivotal role in this paradigm, offering insights into an individual's genetic predisposition to certain diseases and their response to specific therapies. Genomic markers, such as single nucleotide polymorphisms (SNPs), are being investigated to determine their association with increased susceptibility to MI or adverse events post-MI [3]. The integration of pharmacogenomics guides medication selection and dosing based on genetic variants that influence drug metabolism and efficacy. For instance, the cytochrome P450 enzyme CYP2C19 is implicated in the metabolism of clopidogrel, a commonly prescribed antiplatelet medication. Genetic variants in CYP2C19 can impact the drug's effectiveness, leading to variable platelet inhibition and potential treatment failure [4]. Tailoring antiplatelet therapy based on an individual's genotype improves the likelihood of optimal therapeutic outcomes. In addition to pharmacogenomics, biomarker-guided therapy selection is gaining prominence in precision medicine. Biomarkers such as B-type natriuretic peptide (BNP) and N-terminal pro-BNP (NT-proBNP) are indicative of cardiac stress and heart failure. Incorporating these biomarkers into clinical decision-making aids in risk stratification and treatment optimization, particularly in patients with comorbid heart failure [5]. These advancements in diagnostic techniques and precision medicine principles underscore the transformative potential of novel approaches in myocardial infarction management. Part II of this narrative review will delve further into innovative reperfusion strategies, the integration of telemedicine and digital health solutions, and the holistic psychosocial support and rehabilitation programs that are collectively reshaping the landscape of MI care.

## Review

Methods

Literature Search and Selection Criteria

To conduct this narrative review on "Advancements in Myocardial Infarction Management: Exploring Novel Approaches and Strategies," an extensive and systematic literature search was carried out across various databases to ensure comprehensive coverage of relevant studies. The databases utilized for this purpose included PubMed, Medline, Google Scholar, and Web of Science. The search was limited to articles published within the last decade (2013-2023) to capture the latest advancements in myocardial infarction management. A combination of carefully selected keywords was employed to ensure the retrieval of pertinent literature. The primary keywords used in the search strategy were "myocardial infarction," "advancements," "novel approaches," "strategies," "precision medicine," "reperfusion techniques," "telemedicine," and "cardiac rehabilitation." These keywords were chosen to encompass various dimensions of the topic, including emerging strategies, technological innovations, personalized treatments, and holistic patient care.

Inclusion and Exclusion Criteria

The inclusion criteria were established to ensure that the selected studies aligned closely with the scope and objectives of this narrative review. The following inclusion criteria were applied: Studies published within the specified time frame (2013-2023) were considered to capture the most recent advancements. Articles discussing novel approaches and strategies in myocardial infarction management, encompassing aspects such as diagnostics, precision medicine, reperfusion techniques, telemedicine, and rehabilitation, were included. Only peer-reviewed articles, research papers, review articles, and clinical trials were included to ensure a rigorous assessment of the quality and validity of the information. Only articles published in the English language were considered to maintain consistency and facilitate understanding. On the other hand, studies that did not meet the inclusion criteria were excluded. This included articles that focused solely on basic science research, animal studies, or those that lacked relevance to the topic of myocardial infarction management.

Literature Assessment and Synthesis

The selected articles underwent a thorough assessment to extract relevant information and insights. The critical assessment involved evaluating the study design, methodology, sample size, intervention strategies, outcomes measured, and statistical analyses employed. This evaluation helped in discerning the quality and validity of the studies and their applicability to the review's objectives. The synthesized literature was organized thematically to highlight the various advancements in myocardial infarction management. Each thematic section covered specific aspects such as early diagnosis, precision medicine, innovative reperfusion techniques, telemedicine integration, and psychosocial support. The narrative review aimed to not only provide a comprehensive overview of these advancements but also to analyze their potential implications for patient care and outcomes. The synthesis process involved extracting key findings, summarizing study methodologies, and identifying common trends and gaps in the literature. This allowed for the development of a coherent narrative that systematically explored the different dimensions of myocardial infarction management and its recent advancements.

Pathophysiology and etiology of myocardial infarction

MI, commonly known as a heart attack, is a complex and multifaceted cardiovascular event that arises from the disruption of blood flow to a segment of the heart muscle. Understanding the intricate pathophysiological mechanisms and underlying etiological factors that contribute to the occurrence of MI is fundamental to developing effective strategies for its management and prevention. This section delves into the pathophysiology of MI, exploring the mechanisms that underlie its development and addressing the role of genetics, lifestyle factors, and comorbidities in its pathogenesis.

Mechanisms of Myocardial Infarction

The occurrence of MI is primarily attributed to the rupture or erosion of atherosclerotic plaques within the coronary arteries, resulting in the formation of thrombi that obstruct blood flow. Atherosclerosis, a chronic inflammatory process, plays a central role in this pathophysiological cascade. Endothelial dysfunction, characterized by impaired nitric oxide bioavailability and increased expression of adhesion molecules, promotes the recruitment of monocytes and the accumulation of lipids within the arterial wall. These lipid-laden monocytes transform into foam cells, contributing to the formation of fatty streaks, the initial stage of atherosclerotic lesions [5]. Over time, these fatty streaks undergo complex transformations, leading to the development of more advanced plaques characterized by a fibrous cap composed of smooth muscle cells, collagen, and extracellular matrix. Vulnerable plaques are prone to rupture due to the thinning of the fibrous cap and increased inflammation. Plaque rupture exposes the thrombogenic core, triggering platelet aggregation, thrombus formation, and subsequent occlusion of the coronary artery. The cessation of blood flow deprives the downstream myocardium of oxygen and nutrients, resulting in myocardial ischemia and potential necrosis [6].

Genetics and MI Susceptibility

Genetic factors play a pivotal role in the susceptibility to myocardial infarction. Family history of premature coronary artery disease (CAD) is a well-established risk factor, highlighting the influence of genetics in MI predisposition. Genome-wide association studies (GWAS) have identified several genetic variants associated with increased MI risk. For instance, polymorphisms within genes encoding components of the renin-angiotensin-aldosterone system (RAAS) and lipid metabolism pathways have been linked to MI susceptibility [7]. Genetic variations also influence factors such as lipid metabolism, inflammation, and thrombosis, all of which contribute to atherosclerosis and MI. Variants in genes encoding lipoprotein metabolism proteins, such as apolipoprotein E (APOE) and proprotein convertase subtilisin/kexin type 9 (PCSK9), impact lipid levels and, subsequently, atherosclerotic plaque development [8,9]. Polymorphisms in inflammatory cytokine genes, like interleukin-6 (IL-6) and tumor necrosis factor-alpha (TNF-α), modulate inflammatory responses, promoting atherosclerosis progression [9]. Genetic variants affecting platelet function, coagulation factors, and fibrinolytic pathways contribute to thrombotic events that trigger MI [10,11].

Lifestyle Factors and Comorbidities

While genetics plays a significant role, lifestyle factors and comorbidities also substantially contribute to the development of myocardial infarction. The interplay between genetic predisposition and environmental influences underscores the importance of modifiable risk factors in MI prevention. Lifestyle behaviors such as smoking, physical inactivity, unhealthy diet, and excessive alcohol consumption are associated with a higher risk of MI [12]. Obesity and metabolic syndrome are established risk factors for MI, with their prevalence steadily increasing globally. Obesity contributes to a proinflammatory and prothrombotic state, insulin resistance, and dyslipidemia, all of which promote atherosclerosis progression [13]. Diabetes mellitus, characterized by chronic hyperglycemia, augments atherosclerosis development and accelerates plaque vulnerability through mechanisms involving oxidative stress, inflammation, and endothelial dysfunction [13,14]. Hypertension, another key risk factor, exerts mechanical stress on arterial walls, promoting endothelial dysfunction and atherosclerosis initiation [15]. Furthermore, psychological factors such as chronic stress, depression, and social isolation have been linked to increased MI risk through mechanisms involving autonomic dysregulation, inflammatory responses, and unhealthy behaviors [16]. The pathophysiology of myocardial infarction is a complex interplay of genetic predisposition, environmental influences, and underlying comorbidities. Atherosclerosis and plaque rupture are central to the development of MI, with genetics shaping susceptibility to the disease. Lifestyle factors and comorbidities further contribute to the risk of MI through their influence on inflammation, oxidative stress, thrombosis, and atherosclerosis progression. Recognizing these underlying mechanisms is essential in formulating effective strategies for the prevention, diagnosis, and management of myocardial infarction, with a focus on personalized approaches that address both genetic susceptibility and modifiable risk factors.

Early diagnosis and risk stratification

High-Sensitivity Biomarkers

Biomarkers play a pivotal role in the early diagnosis and risk stratification of MI. High-sensitivity biomarkers have emerged as valuable tools in this regard. One of the most widely studied biomarkers is cardiac troponin (cTn). Troponin is a protein complex found in cardiac muscle, and its release into the bloodstream is indicative of myocardial injury. High-sensitivity troponin assays allow for the detection of even minor cardiac muscle damage, enabling the early diagnosis of MI. High-sensitivity cTn assays have significantly improved the accuracy and speed of MI diagnosis. They can detect cTn levels within a few hours of symptom onset, compared to older assays that required several days to provide reliable results. The European Society of Cardiology (ESC) and the American College of Cardiology (ACC) guidelines recommend high-sensitivity cTn testing for diagnosing MI, underscoring its importance in clinical practice [15]. Another promising biomarker is the heart-type fatty acid-binding protein (H-FABP), which is released into the bloodstream rapidly after myocardial injury. H-FABP has shown promise in the early diagnosis of MI, particularly in the early hours following symptom onset. Combining high-sensitivity cTn and H-FABP measurements may enhance the diagnostic accuracy of MI, facilitating rapid triage and treatment [16]. In addition to troponin and H-FABP, other biomarkers, such as myoglobin, creatine kinase-MB (CK-MB), and natriuretic peptides, are also used for risk stratification and assessing the extent of myocardial injury. These high-sensitivity biomarkers provide clinicians with a comprehensive panel to aid in the early diagnosis and risk stratification of MI.

Advanced Imaging Techniques

Advanced imaging techniques have revolutionized the field of cardiology, allowing for precise visualization of cardiac structures and function. These techniques are crucial in the early diagnosis of MI and risk stratification. Two of the most notable advancements in this area are cardiac MRI and coronary computed tomography angiography (CCTA). Cardiac MRI is a non-invasive imaging modality that offers high spatial resolution and excellent tissue characterization. It can provide detailed information about myocardial viability, perfusion, and function. In the context of MI diagnosis, cardiac MRI can help identify areas of myocardial infarction, assess myocardial edema in the acute phase, and quantify ventricular function, which is crucial for risk stratification [17]. CCTA is another powerful imaging tool that has gained prominence in recent years. CCTA allows for the visualization of coronary arteries and the assessment of coronary stenosis. This technique is particularly useful for ruling out CAD in patients with suspected MI and low-to-intermediate pre-test probability. CCTA can rapidly identify obstructive CAD and guide therapeutic decisions, reducing the time required for diagnosis and treatment [18]. Moreover, emerging imaging techniques such as positron emission tomography (PET) and 3D echocardiography hold promise in providing additional information for risk stratification and monitoring of MI patients. These advanced imaging modalities contribute to the early diagnosis of MI and play a vital role in assessing patient risk by providing a comprehensive evaluation of cardiac structure and function.

Risk Prediction Models

Risk prediction models have become indispensable tools in clinical practice for the stratification of MI patients. These models use a combination of clinical variables, biomarker measurements, and imaging data to estimate the probability of adverse outcomes following MI, such as recurrent MI, heart failure, or death. Two widely recognized risk prediction models are the Global Registry of Acute Coronary Events (GRACE) and the Thrombolysis in Myocardial Infarction (TIMI) risk scores. The GRACE risk score incorporates variables such as age, heart rate, systolic blood pressure, creatinine level, Killip class, cardiac arrest at admission, ST-segment deviation on the initial ECG, elevated cardiac biomarkers, and previous MI or heart failure. It provides an estimate of the patient's risk of death and adverse cardiovascular events at various time points following MI [19]. The TIMI risk score, on the other hand, assesses the patient's risk of death, recurrent MI, or severe recurrent ischemia requiring revascularization. It considers factors such as age, diabetes, hypertension, prior angina, prior coronary artery bypass grafting or percutaneous coronary intervention, and the number of coronary artery stenoses greater than 50%. The TIMI risk score is particularly valuable in guiding treatment decisions, such as the need for invasive coronary angiography and revascularization procedures [20]. Recent advancements in risk prediction models involve the incorporation of genetic information, machine learning algorithms, and artificial intelligence (AI) techniques. These approaches aim to enhance the accuracy of risk stratification by considering a broader range of factors and exploiting large datasets to identify hidden patterns and associations. Machine learning models, such as random forests and deep neural networks, have shown promise in predicting post-MI outcomes based on clinical, genetic, and imaging data [21]. Early diagnosis and risk stratification are critical components of managing myocardial infarction effectively. High-sensitivity biomarkers, advanced imaging techniques, and risk prediction models have transformed the landscape of MI diagnosis and patient risk assessment. High-sensitivity troponin assays and other biomarkers enable rapid detection of myocardial injury, aiding in early diagnosis. Advanced imaging modalities like cardiac MRI and CCTA provide comprehensive insights into cardiac structure and function, facilitating risk stratification and treatment decisions. Risk prediction models like GRACE and TIMI scores help clinicians estimate the likelihood of adverse outcomes following MI, guiding personalized care [21,22]. As research continues to advance, we can expect further refinements in these diagnostic and risk stratification tools. Integrating genetic information and harnessing the power of artificial intelligence may unlock new insights and improve the precision of risk assessment. Ultimately, these advancements contribute to better patient outcomes, reduced morbidity and mortality, and a more efficient healthcare system.

Precision medicine in myocardial infarction management

Precision medicine, also known as personalized medicine, represents a transformative approach to healthcare that takes into account individual variability in genes, environment, and lifestyle when tailoring medical decisions and treatments. This concept has gained significant momentum in the management of various diseases, including MI. Myocardial infarction, commonly known as a heart attack, is a complex cardiovascular condition with diverse underlying mechanisms. Precision medicine in MI management involves the use of genetic profiling, pharmacogenomics, and biomarker-guided therapies to develop personalized treatment strategies. Additionally, novel pharmaceutical agents, regenerative therapies, and interventions targeting specific molecular pathways are emerging as promising approaches to improve outcomes in MI patients.

Genetic Profiling in MI Management

Genetic profiling plays a central role in precision medicine for MI management. Genetic variations can influence an individual's susceptibility to MI, response to therapy, and risk of complications. Several genes have been implicated in MI risk, including those involved in lipid metabolism (e.g., PCSK9), inflammation (e.g., IL-6), and blood clotting (e.g., factor V Leiden). Genetic testing allows for the identification of these variations and enables risk stratification. One well-known genetic factor associated with MI is the APOE gene. Different APOE genotypes have varying effects on lipid metabolism and, consequently, the risk of atherosclerotic plaque formation. Individuals carrying the APOE ε4 allele have an increased risk of hypercholesterolemia and coronary artery disease, making them suitable candidates for more aggressive lipid-lowering therapies [22].

Pharmacogenomics in Myocardial Infarction Management

Pharmacogenomics is the study of how an individual's genetic makeup influences their response to drugs. In the context of MI management, pharmacogenomics plays a crucial role in tailoring medication regimens to optimize treatment outcomes while minimizing adverse effects. One of the most prominent examples in this field is the use of clopidogrel, an antiplatelet drug commonly prescribed after MI. Clopidogrel is a prodrug that requires activation by the enzyme CYP2C19 to become active and inhibit platelet aggregation effectively. However, individuals with certain CYP2C19 genetic variants metabolize clopidogrel less efficiently, leading to reduced drug efficacy and an increased risk of recurrent cardiovascular events. Pharmacogenomic testing can identify patients with these genetic variants and guide the selection of alternative antiplatelet therapies, such as prasugrel or ticagrelor, which do not depend on CYP2C19 for activation [23].

Biomarker-Guided Therapies

Biomarkers are measurable substances in the body that provide information about a disease or its progression. In MI management, several biomarkers are used to guide treatment decisions and assess prognosis. One such biomarker is high-sensitivity cardiac troponin (hs-cTn), which is released into the bloodstream during myocardial injury. The levels of hs-cTn can help diagnose MI and stratify patients based on the extent of myocardial damage. Biomarker-guided therapies involve tailoring treatment based on specific biomarker profiles. For example, in patients with non-ST-segment elevation myocardial infarction (NSTEMI), elevated hs-cTn levels may indicate a higher risk of adverse cardiovascular events. These patients may benefit from more aggressive interventions, such as early coronary angiography and revascularization, compared to those with lower hs-cTn levels [24].

Novel Pharmaceutical Agents

Precision medicine in MI management extends to the development of novel pharmaceutical agents that target specific molecular pathways involved in the disease. These agents aim to provide more effective and personalized treatments for MI patients. One such example is the use of PCSK9 inhibitors, which have revolutionized lipid-lowering therapy. PCSK9 is a protein that regulates the levels of low-density lipoprotein cholesterol (LDL-C) in the blood by promoting the degradation of the LDL receptor. Mutations in the PCSK9 gene can lead to familial hypercholesterolemia and a significantly increased risk of premature MI. PCSK9 inhibitors, such as evolocumab and alirocumab, work by blocking the action of PCSK9, resulting in a substantial reduction in LDL-C levels. These agents are particularly valuable for individuals with familial hypercholesterolemia and those who do not achieve target LDL-C levels with standard therapies [25].

Regenerative Therapies

Regenerative therapies represent a cutting-edge approach in precision medicine for MI management. These therapies aim to repair damaged cardiac tissue and improve heart function by stimulating the regeneration of cardiomyocytes. One promising avenue of research involves the use of stem cells, such as induced pluripotent stem cells (iPSCs) and cardiac progenitor cells (CPCs). iPSCs are reprogrammed adult cells with the potential to differentiate into various cell types, including cardiomyocytes. Researchers have explored the transplantation of iPSC-derived cardiomyocytes into the hearts of MI patients to replace damaged tissue. This approach holds great promise for myocardial regeneration and has shown encouraging results in preclinical studies [26]. Another regenerative approach involves the use of CPCs, which are resident cardiac stem cells capable of differentiating into cardiomyocytes. CPC-based therapies aim to enhance the endogenous regenerative capacity of the heart. Clinical trials are ongoing to assess the safety and efficacy of CPC transplantation in MI patients, with the hope of improving cardiac function and reducing post-MI complications [27].

Interventions Targeting Specific Molecular Pathways

Advances in molecular biology and our understanding of the molecular pathways involved in MI have paved the way for interventions that target these specific pathways. One such pathway is the inflammasome, a multiprotein complex involved in the inflammatory response following myocardial injury. Excessive inflammasome activation can exacerbate tissue damage and contribute to adverse remodeling of the heart. Several experimental drugs are being developed to target the inflammasome pathway in MI. These drugs aim to modulate the inflammatory response and prevent excessive inflammation-induced damage. Clinical trials are underway to evaluate the safety and efficacy of these interventions, with the hope of reducing post-MI inflammation and improving outcomes [28]. Precision medicine has ushered in a new era in the management of myocardial infarction. Genetic profiling, pharmacogenomics, and biomarker-guided therapies allow for personalized treatment approaches that take into account individual variability. Novel pharmaceutical agents, regenerative therapies, and interventions targeting specific molecular pathways hold the promise of improving outcomes in MI patients by addressing the underlying mechanisms of the disease. As research in precision medicine continues to advance, it is crucial to integrate these approaches into clinical practice to benefit a broader range of patients. By tailoring treatments to each individual's unique genetic and molecular profile, healthcare providers can optimize therapeutic outcomes and ultimately reduce the burden of myocardial infarction on public health.

Innovative reperfusion techniques

Reperfusion therapy is the cornerstone of MI management, aimed at restoring blood flow to the ischemic myocardium and salvaging viable cardiac tissue. While conventional reperfusion techniques, such as primary PCI and fibrinolytic therapy, have significantly improved outcomes for MI patients, ongoing research and technological advancements have led to innovative approaches that further optimize reperfusion outcomes and minimize complications [29]. In this discussion, we will explore three innovative reperfusion techniques: distal embolic protection devices, bioresorbable scaffolds, and microvascular interventions, elucidating how these techniques are reshaping the landscape of MI management.

Distal Embolic Protection Devices

Distal embolic protection devices (EPDs) represent a groundbreaking development in the field of interventional cardiology, particularly in the context of acute coronary syndromes (ACS). These devices are designed to prevent the downstream embolization of atherothrombotic debris during PCI procedures, which can compromise microvascular flow and lead to adverse clinical outcomes. The primary mechanism of action of EPDs involves the capture and retrieval of embolic debris, including thrombi and atheromatous plaque, from the distal coronary circulation. One of the most widely used EPDs is the FilterWire Embolic Protection System, which employs a filter-like device that captures and entraps debris while allowing uninterrupted blood flow [29]. This mechanism reduces the risk of distal embolization and microvascular injury during PCI. The use of EPDs has been shown to be particularly beneficial in patients with complex lesion morphology, high thrombus burden, and vulnerable plaques. Clinical trials, such as the EMERALD study, have demonstrated the safety and efficacy of EPDs in improving myocardial perfusion, reducing infarct size, and lowering the risk of major adverse cardiac events [30].

Bioresorbable Scaffolds

Bioresorbable scaffolds (BRS) represent a revolutionary advancement in coronary stent technology. Unlike conventional metallic stents, BRS are designed to provide temporary support to the coronary artery while gradually resorbing over time, leaving behind a healed vessel with restored vasomotion and physiological function. This innovative approach aims to address some of the limitations associated with permanent metallic stents, such as late stent thrombosis and impaired vasomotion. One of the most extensively studied BRS is the Absorb Bioresorbable Vascular Scaffold (BVS). Made of a biocompatible polymer, the scaffold provides mechanical support to the coronary artery during the initial healing phase and drug elution to prevent restenosis. As the polymer gradually resorbs, the vessel regains its natural structure and function. Clinical trials, including ABSORB III and ABSORB IV, have assessed the safety and efficacy of BVS in comparison to metallic drug-eluting stents [31]. These trials have demonstrated similar rates of target lesion failure between BVS and metallic stents at short-term follow-up. However, BVS has shown a unique advantage in the restoration of vasomotion and the reduction of late adverse events, although longer-term data are still being evaluated.

Microvascular Interventions

The microcirculation plays a critical role in myocardial perfusion and recovery following reperfusion therapy. Microvascular dysfunction, characterized by impaired flow and increased resistance in the microvessels, can lead to poor outcomes in MI patients, including no-reflow phenomenon and adverse left ventricular remodeling. Innovative microvascular interventions have emerged to address these challenges and optimize microvascular function. One promising microvascular intervention is the use of vasodilators, such as adenosine and nicorandil, during PCI procedures. These agents work by relaxing the smooth muscle in the microvessels, improving microvascular flow and reducing the risk of no-reflow [32]. Additionally, intracoronary administration of glycoprotein IIb/IIIa inhibitors, such as abciximab, has been shown to reduce microvascular obstruction and improve myocardial perfusion in selected cases [25]. Another innovative approach to microvascular intervention involves the use of intracoronary imaging techniques, such as intravascular ultrasound (IVUS) and optical coherence tomography (OCT). These imaging modalities provide high-resolution visualization of the coronary arteries and microvasculature, allowing for the assessment of plaque burden, stent apposition, and microvascular function [20]. IVUS-guided PCI, in particular, has been associated with improved outcomes and reduced rates of major adverse cardiovascular events [21]. Furthermore, the development of microvascular-specific therapies, such as adenosine receptor agonists and microvascular embolization prevention strategies, holds promise for mitigating microvascular injury and improving outcomes in MI patients. These interventions are currently under investigation in clinical trials and may offer new avenues for personalized MI management.

Optimizing Reperfusion Outcomes and Minimizing Complications

The innovative reperfusion techniques discussed above offer several advantages in optimizing reperfusion outcomes and minimizing complications in MI management. First, distal embolic protection devices help reduce the risk of distal embolization during PCI procedures, which is associated with microvascular injury and adverse clinical outcomes. By capturing and removing embolic debris, EPDs promote better myocardial perfusion and limit the extent of myocardial damage. Second, bioresorbable scaffolds address the limitations of permanent metallic stents by providing temporary support to the coronary artery. As the scaffold gradually resorbs, the vessel's natural function is restored, reducing the risk of late stent thrombosis and improving long-term outcomes. Third, microvascular interventions, such as vasodilators and intracoronary imaging techniques, target microvascular dysfunction, a critical factor in myocardial reperfusion. These interventions improve microvascular flow, reduce the risk of no-reflow, and enhance myocardial recovery [29]. In summary, these innovative reperfusion techniques offer a personalized approach to MI management by addressing specific patient needs and characteristics. While further research and long-term data are needed to establish their full clinical potential, these advancements hold great promise in reshaping the landscape of MI treatment, ultimately improving patient outcomes and reducing the burden of this devastating condition.

Reperfusion therapy is the cornerstone of MI management, aimed at restoring blood flow to the ischemic myocardium and salvaging viable cardiac tissue. While conventional reperfusion techniques, such as primary PCI and fibrinolytic therapy, have significantly improved outcomes for MI patients, ongoing research and technological advancements have led to innovative approaches that further optimize reperfusion outcomes and minimize complications [30]. In this discussion, we will explore three innovative reperfusion techniques: distal embolic protection devices, bioresorbable scaffolds, and microvascular interventions, elucidating how these techniques are reshaping the landscape of MI management.

Integration of telemedicine and digital health in post-myocardial infarction care: revolutionizing remote patient monitoring

MI, commonly known as a heart attack, is a life-threatening event that requires ongoing medical management and close monitoring to prevent recurrent cardiovascular events and complications. The integration of telemedicine and digital health solutions has emerged as a transformative approach in post-MI care. This integration encompasses remote patient monitoring, wearable devices, and mobile applications, all of which facilitate real-time data exchange, enhance patient engagement, and enable timely interventions. In this discussion, we will delve into the evolving landscape of post-MI care, exploring the innovative ways telemedicine and digital health are revolutionizing patient management.

Remote Patient Monitoring

Remote patient monitoring (RPM) is a fundamental component of post-MI care through telemedicine and digital health. RPM involves the continuous collection and transmission of patient data from home or other non-clinical settings to healthcare providers. This real-time data exchange enables clinicians to monitor patients' vital signs, medication adherence, and disease progression, thus facilitating early intervention and personalized care. One of the critical parameters monitored in post-MI care is blood pressure. Elevated blood pressure is a significant risk factor for recurrent cardiovascular events, making its control crucial. Digital blood pressure monitors, connected to telehealth platforms, allow healthcare providers to remotely monitor patients' blood pressure trends and adjust medications accordingly. A study published in JAMA Internal Medicine demonstrated that RPM combined with telemonitoring significantly improved blood pressure control and medication adherence in post-MI patients compared to traditional care [30]. Another vital aspect of RPM is the continuous monitoring of ECG data. Advances in wearable ECG devices, such as smartwatches and patches, enable patients to record and transmit ECG data to their healthcare providers in real time. This technology allows for the early detection of arrhythmias or ST-segment changes, enabling prompt intervention. For example, the Apple Watch's ECG feature has been shown to accurately detect atrial fibrillation, a common arrhythmia associated with post-MI complications [31].

Wearable Devices

Wearable devices have gained immense popularity in recent years and are increasingly integrated into post-MI care. These devices, which include smartwatches, fitness trackers, and health monitoring devices, offer several advantages in monitoring patients' health and promoting active lifestyles. Smartwatches equipped with heart rate sensors and ECG capabilities can continuously monitor heart rhythms and detect irregularities. In addition to detecting arrhythmias, they can also monitor physical activity, sleep patterns, and stress levels, all of which are essential considerations in post-MI care. Patients can share this data with their healthcare providers, enabling timely interventions and personalized treatment plans. Moreover, wearable devices with GPS functionality can track patients' locations and activities, which is especially valuable for patients at risk of recurrent cardiovascular events. If a patient experiences symptoms suggestive of a recurrent MI, such as chest pain or shortness of breath, the wearable device can automatically alert emergency services and provide the patient's location. This feature can significantly reduce the time it takes for patients to receive medical attention, potentially saving lives [28].

Mobile Applications

Mobile applications, commonly referred to as "apps," have become indispensable tools in post-MI care. These apps offer a wide range of functionalities, from medication reminders and symptom tracking to virtual visits with healthcare providers. Their user-friendly interfaces and accessibility on smartphones make them convenient tools for both patients and healthcare professionals. Medication adherence is a critical component of post-MI care, as prescribed medications help prevent recurrent cardiovascular events [31]. Mobile apps can provide patients with medication reminders and educational resources about their medications, improving adherence rates. Additionally, some apps can interact with wearable devices to track medication ingestion, providing real-time feedback to both patients and healthcare providers. Symptom tracking is another essential aspect of post-MI care. Patients can use mobile apps to log symptoms such as chest pain, shortness of breath, or fatigue. These apps can provide trend analyses and alert healthcare providers if symptoms worsen or new symptoms emerge. This real-time symptom tracking allows for early intervention and timely adjustments to treatment plans. Virtual visits through mobile apps have become increasingly popular, especially in the context of telemedicine [32]. Patients can schedule virtual appointments with their healthcare providers, reducing the need for in-person visits and the associated travel and waiting times. During these virtual visits, providers can review patient data from wearable devices and mobile apps, discuss symptoms, and adjust treatment plans as needed. This approach not only enhances patient convenience but also reduces the risk of exposure to infectious diseases, an important consideration in the current healthcare landscape.

Enhancing Patient Engagement

One of the significant advantages of integrating telemedicine and digital health in post-MI care is the enhancement of patient engagement. Patients become active participants in their care, as they have access to their health data, educational resources, and tools for self-management [33]. Patient engagement is further facilitated through features such as secure messaging with healthcare providers, online support groups, and personalized health recommendations based on the data collected by wearable devices and mobile apps. Patients are more likely to adhere to their treatment plans and make positive lifestyle changes when they are engaged in their care and have the support of healthcare professionals and fellow patients.

Timely Interventions and Personalized Care

The integration of telemedicine and digital health in post-MI care allows for timely interventions and personalized care plans. Healthcare providers can remotely monitor patients' vital signs, review ECG data, track symptoms, and assess medication adherence in real time. When deviations from the norm are detected, providers can promptly reach out to patients to discuss their symptoms, adjust medications, or schedule virtual visits [33]. Personalized care plans are tailored to each patient's unique needs and health status. For example, a patient who is consistently active and adherent to their medication regimen may require less frequent monitoring and follow-up, whereas a patient with multiple risk factors and a history of poor adherence may benefit from more intensive interventions and support.

Psychosocial support and cardiac rehabilitation in myocardial infarction recovery

MI is a life-altering event with far-reaching physical and emotional consequences. Beyond the medical interventions, the journey to recovery after an MI is greatly influenced by psychosocial factors. This discussion will explore the critical importance of psychosocial support and cardiac rehabilitation in MI recovery. We will delve into psychological counseling, stress management, lifestyle interventions, and how structured cardiac rehabilitation programs contribute to improved patient outcomes.

Psychosocial Impact of Myocardial Infarction

The psychological impact of an MI is profound and can affect patients in various ways. Patients often experience a range of emotions, including fear, anxiety, depression, anger, and grief. These emotional responses can be triggered by the life-threatening nature of the event, uncertainty about the future, and concerns about physical limitations and dependence on medications. One of the primary psychological challenges faced by MI survivors is the fear of recurrent events [33]. The fear of another heart attack can be overwhelming, leading to hypervigilance about physical sensations and a constant state of anxiety. These fears can have a detrimental impact on patients' quality of life and overall well-being. Depression is another common psychological consequence of MI. The biological and psychological links between heart disease and depression are well-established. Depressive symptoms can lead to reduced adherence to medical therapies, poor lifestyle choices (such as unhealthy eating habits and physical inactivity), and an increased risk of recurrent cardiovascular events [34].

Psychosocial Support and Psychological Counseling

Psychosocial support is a crucial component of MI recovery. It encompasses various interventions aimed at addressing the emotional and psychological needs of patients. One essential element of psychosocial support is psychological counseling. Psychological counseling provides a safe and confidential space for patients to express their fears, anxieties, and emotional challenges related to their MI. Cognitive-behavioral therapy (CBT), for instance, is a well-established therapeutic approach that helps patients identify negative thought patterns and develop coping strategies to manage anxiety and depression. Additionally, counseling can assist patients in understanding the emotional aspects of their condition, including the grief associated with lifestyle changes and the sense of loss of their pre-MI self. It can also address issues related to body image and self-esteem, which may be affected by physical changes resulting from the MI or its treatment [34].

Stress Management

Stress management is a critical aspect of MI recovery because chronic stress can exacerbate cardiovascular risk factors and increase the likelihood of recurrent events. Patients who have experienced an MI are often encouraged to adopt stress-reduction strategies. One effective stress management technique is mindfulness-based stress reduction (MBSR). MBSR incorporates mindfulness meditation, deep breathing exercises, and yoga to help patients become more aware of their thoughts and emotions and develop healthier responses to stressors. Research has shown that MBSR can lead to improvements in psychological well-being and a reduction in cardiovascular risk factors, such as blood pressure and inflammation [34].

Lifestyle Interventions

Lifestyle interventions are a cornerstone of MI recovery and are closely intertwined with psychosocial support. Following an MI, patients are encouraged to make significant changes to their lifestyle to reduce cardiovascular risk factors and improve overall health. Dietary modifications are a crucial component of lifestyle interventions. A heart-healthy diet, rich in fruits, vegetables, whole grains, lean proteins, and healthy fats, can help control blood pressure, cholesterol levels, and body weight [35]. Psychosocial support can play a role in helping patients make sustainable dietary changes by addressing emotional eating patterns and food-related stressors [34]. Physical activity is another critical lifestyle intervention. Regular exercise can improve cardiovascular fitness, reduce stress, and enhance overall well-being. However, some patients may experience anxiety or fear about engaging in physical activity post-MI. Psychosocial support can provide guidance and motivation to help patients gradually incorporate exercise into their routine [35]. Smoking cessation is of paramount importance in MI recovery. Smoking is a major risk factor for cardiovascular disease, and quitting smoking is associated with a significant reduction in the risk of recurrent events. Psychosocial support, including counseling and support groups, can be instrumental in helping patients overcome nicotine addiction.

Structured Cardiac Rehabilitation Programs

Structured cardiac rehabilitation programs are comprehensive interventions designed to support the recovery and rehabilitation of MI patients. These programs typically involve a multidisciplinary team, including cardiologists, nurses, dietitians, exercise physiologists, and psychologists, working together to address the physical, psychological, and social aspects of recovery. Cardiac rehabilitation programs typically consist of the following components. Supervised exercise sessions tailored to the patient's individual fitness level are a central element of cardiac rehabilitation [30]. These sessions aim to improve cardiovascular fitness, reduce risk factors, and enhance overall physical health. Patients receive guidance on adopting heart-healthy lifestyle habits, including dietary changes, smoking cessation, and stress management. Patients learn about their condition, medications, risk factors, and strategies for self-care. This education empowers patients to take an active role in managing their health. Cardiac rehabilitation programs often include psychological counseling and support to address the emotional challenges associated with MI recovery. The team monitors and manages cardiovascular risk factors, such as blood pressure, cholesterol levels, and blood sugar, to reduce the risk of recurrent events. Patients have the opportunity to connect with others who have experienced similar challenges through support groups and peer interactions. Structured cardiac rehabilitation programs have been shown to have numerous benefits for MI patients. Research indicates that participation in cardiac rehabilitation is associated with a reduction in cardiovascular mortality, hospital readmissions, and improved quality of life [35].

Psychosocial support and cardiac rehabilitation play integral roles in the recovery journey of MI patients. The psychosocial impact of an MI can be profound, affecting emotions, behaviors, and overall well-being. Addressing these psychosocial factors through psychological counseling, stress management, and lifestyle interventions is essential for improving patient outcomes and enhancing their quality of life. Structured cardiac rehabilitation programs offer a comprehensive approach to MI recovery, addressing not only physical health but also psychological and social well-being [36]. These programs empower patients to make meaningful lifestyle changes, reduce cardiovascular risk factors, and actively participate in their recovery. As healthcare providers continue to recognize the importance of psychosocial support and cardiac rehabilitation in MI care, it is crucial to ensure that these services are accessible and tailored to the individual needs of patients. By addressing the holistic needs of MI survivors, healthcare professionals can contribute significantly to their long-term health and well-being.

Discussion and implications: advancements in myocardial infarction management

This comprehensive discussion has explored several critical advancements in the management of MI, ranging from early diagnosis and risk stratification to precision medicine, innovative reperfusion techniques, and the integration of telemedicine and digital health. Each section has provided insights into how these advancements are reshaping the landscape of MI care. In this section, we will summarize the key findings from each section and discuss the implications of these advancements for patient care, outcomes, and the healthcare system. Additionally, we will highlight potential challenges and areas for further research. The early diagnosis and risk stratification of MI have significantly evolved with the emergence of high-sensitivity biomarkers, advanced imaging techniques, and risk prediction models. High-sensitivity troponin assays have revolutionized the diagnosis of MI, enabling the detection of myocardial injury even in its earliest stages. Advanced imaging modalities, such as cardiac MRI and CCTA, provide detailed information about coronary anatomy and myocardial viability. Risk prediction models, incorporating clinical, imaging, and biomarker data, offer a more accurate assessment of patient risk [2]. Early diagnosis allows for prompt initiation of reperfusion therapy and risk-tailored interventions, improving patient outcomes. It also contributes to healthcare resource utilization efficiency by preventing unnecessary hospitalizations. The increased sensitivity of high-sensitivity troponin assays can lead to more false-positive results, necessitating careful interpretation. Additionally, the adoption of advanced imaging techniques may require specialized training and resources. Further research is needed to refine risk prediction models, incorporating novel biomarkers and imaging parameters. Additionally, investigations into the cost-effectiveness of these diagnostic advancements are warranted. Precision medicine in MI management involves genetic profiling, pharmacogenomics, and biomarker-guided therapies. Genetic profiling allows for the identification of genetic variants associated with MI risk and drug response. Pharmacogenomics tailors medication regimens to individual genetic profiles, enhancing drug efficacy and minimizing adverse effects. Biomarker-guided therapies, such as antiplatelet therapy optimization based on platelet function testing, offer personalized treatment approaches [4]. Precision medicine holds the promise of optimizing treatment outcomes by tailoring therapies to individual patient characteristics, minimizing adverse events, and improving medication adherence. The integration of genetic testing into routine clinical practice may face challenges related to cost, accessibility, and ethical considerations. Interpretation of genetic data and its clinical relevance also require ongoing research and guidance. Further research is needed to identify additional genetic markers associated with MI risk and drug response. Additionally, studies evaluating the long-term clinical impact and cost-effectiveness of precision medicine approaches are warranted. Innovative reperfusion techniques beyond conventional strategies, such as distal embolic protection devices, bioresorbable scaffolds, and microvascular interventions, have been discussed. These techniques optimize reperfusion outcomes by reducing distal embolization, promoting vessel healing, and addressing microvascular dysfunction [29,30]. Innovative reperfusion techniques offer personalized approaches to MI management, reducing complications and improving patient outcomes. They have the potential to enhance the success of reperfusion therapy and minimize the extent of myocardial damage. Wider adoption of these innovative techniques may require specialized training for healthcare providers and increased procedural costs. Long-term data on the safety and efficacy of these approaches are also needed. Ongoing research should focus on refining these techniques, assessing their long-term outcomes, and identifying patient populations that may benefit most from them. The integration of telemedicine and digital health solutions in post-MI care, including remote patient monitoring, wearable devices, and mobile applications, has been explored. These technologies enable real-time data exchange, enhance patient engagement, and facilitate timely interventions [33]. Telemedicine and digital health solutions empower patients to actively participate in their post-MI care, improve medication adherence, and enable early detection of complications. They also offer the potential for more convenient and accessible healthcare delivery. Challenges related to data security, privacy, and health disparities must be addressed. Not all patients may have access to the necessary technology, potentially exacerbating healthcare inequalities. Further research should focus on the long-term impact of telemedicine and digital health solutions on patient outcomes and healthcare utilization. Additionally, studies on strategies to address healthcare disparities in the adoption of these technologies are crucial. Collectively, these advancements in MI management have the potential to transform patient care and improve outcomes. Early diagnosis and risk stratification enable timely interventions, reducing morbidity and mortality. Precision medicine tailors treatments to individual patients, optimizing therapy effectiveness and safety. Innovative reperfusion techniques minimize complications and enhance myocardial salvage. Integration of telemedicine and digital health solutions enhances patient engagement, facilitates self-management, and enables proactive care. Incorporating these advancements into routine clinical practice can lead to more efficient healthcare delivery. It can reduce hospital readmissions, lower healthcare costs, and improve patients' quality of life. Moreover, the shift towards personalized medicine and remote monitoring aligns with the broader goals of healthcare systems to provide patient-centered, value-based care [34,35]. Despite these promising advancements, challenges remain. Ensuring equitable access to these innovations and addressing healthcare disparities is essential. Additionally, research is needed to validate the long-term benefits, cost-effectiveness, and safety profiles of these approaches. The ethical implications of genetic testing and data privacy in digital health also warrant ongoing consideration. Furthermore, the integration of these advancements into clinical practice requires healthcare providers to adapt to new technologies and treatment paradigms. Education and training programs should be developed to support this transition.

## Conclusions

Recent advancements in MI management signify a profound departure from conventional approaches, ushering in a new era of personalized, holistic, and patient-centered care. These innovations, encompassing early diagnosis and risk stratification, precision medicine, innovative reperfusion techniques, and telemedicine integration, constitute a transformative shift in MI management. Key among these changes is the prioritization of early diagnosis and risk assessment. High-sensitivity biomarkers, advanced imaging, and risk prediction models have revolutionized MI identification, facilitating rapid intervention to limit myocardial damage and improve patient outcomes. This transition from a one-size-fits-all approach to precision medicine ensures treatments align with individual risk profiles, enhancing effectiveness and safety. Precision medicine plays a pivotal role in these advances, enabling tailored treatment plans based on patients' unique genetic and clinical characteristics. Genetic profiling, pharmacogenomics, and biomarker-guided therapies optimize drug regimens, reduce adverse effects, and boost patient adherence, ultimately enhancing long-term outcomes and quality of life. Innovative reperfusion techniques, like distal embolic protection devices and bioresorbable scaffolds, underscore the shift towards personalized care. These techniques address specific patient needs, minimizing complications and optimizing reperfusion outcomes, leading to improved cardiac function and a better quality of life for survivors. The integration of telemedicine and digital health solutions signifies a holistic approach to post-MI care. Remote monitoring, wearables, and mobile apps empower patients to actively engage in their recovery. Real-time data exchange enhances patient engagement, enables early intervention, and supports self-management, offering accessible healthcare options that improve patient quality of life and may help reduce healthcare disparities. Overall, recent MI management advancements prioritize early detection, personalized treatment, and comprehensive support, encompassing psychosocial and lifestyle factors. This approach not only reduces the MI burden by preventing complications and recurrent events but also elevates survivors' overall quality of life.
